# USMLE step 1 and step 2 CK as indicators of resident performance

**DOI:** 10.1186/s12909-023-04530-8

**Published:** 2023-07-31

**Authors:** Conner V. Lombardi, Neejad T. Chidiac, Benjamin C. Record, Jeremy J. Laukka

**Affiliations:** 1 University of Toledo College of Medicine and Life Sciences, Toledo, Ohio United States; 2 Departments of Medical Education and Neurology, University of Toledo College of Medicine and Life Sciences, Toledo, Ohio United States

**Keywords:** USMLE, NBME, Step 1, Step 2, Standardized Testing, Medical Education

## Abstract

**Background:**

The purpose of this systematic review was to (1) determine the scope of literature measuring USMLE Step 1 and Step 2 CK as predictors or indicators of quality resident performance across all medical specialties and (2) summarize the ability of Step 1 and Step 2 CK to predict quality resident performance, stratified by ACGME specialties, based on available literature.

**Methods:**

This systematic review was designed according to the Preferred Reporting Items for Systematic Reviews and Meta-Analyses (PRISMA) [16]. The original search strategy surveyed MEDLINE and was adapted to survey Cochrane Library and Embase. A study was deemed eligible if it provided all three of the following relevant information: (a) Step 1 or Step 2 CK as indicators for (b) resident outcomes in (c) any ACGME accredited specialty training program.

**Results:**

A total of 1803 articles were screened from three separate databases. The 92 included studies were stratified by specialty, with Surgery (21.7% [20/92]), Emergency Medicine (13.0% [12/92]), Internal Medicine (10.9% [10/92]), and Orthopedic Surgery (8.7% [8/92]) being the most common. Common resident performance measures included ITE scores, board certification, ACGME milestone ratings, and program director evaluations.

**Conclusions:**

Further studies are imperative to discern the utility of Step 1 and Step 2 CK as predictors of resident performance and as tools for resident recruitment and selection. The results of this systematic review suggest that a scored Step 1 dated prior to January 2022 can be useful as a tool in a holistic review of future resident performance, and that Step 2 CK score performance may be an effective tool in the holistic review process. Given its inherent complexity, multiple tools across many assessment modalities are necessary to assess resident performance comprehensively and effectively.

**Supplementary Information:**

The online version contains supplementary material available at 10.1186/s12909-023-04530-8.

## Introduction

In the early 1990s, the birth and evolution of the United States Medical Licensing Examination (USMLE) was implemented as the defining pathway for medical licensure in the United States [1]. It currently consists of Step 1, assessing the application of foundational sciences; Step 2 Clinical Knowledge (CK), assessing acquired knowledge of clinical medicine; and Step 3, assessing knowledge of clinical medicine and patient management. The landscape of medical education has undergone transformational change resulting in a growing number of schools strategically moving Step 1 following core clerkships education [2] followed by Step 2 CK which is traditionally completed during the fourth year of medical school [3]. The USMLE announced in January 2021, that Step 2 Clinical Skills (CS) would be indefinitely canceled citing the initial postponement during the COVID-19 pandemic and the ever-changing environment of medical education [4]. USMLE Step 3 is the final examination, and it is commonly taken during the PGY-1 year of residency and assesses competency in clinical knowledge and skills imperative for the unsupervised practice of medicine as a physician [5].

The USMLE Step 1 and Step 2 CK exams together have been two of the most important and influential factors for Residency Program Directors when assessing medical students’ candidacy for all residency training programs in the United States [6–9]. As of January 2022, Step 1 transitioned to a pass/fail score structure, reflecting its original purpose of being a criterion-referenced examination that determines whether examinees meet a pre-defined knowledge standard. Its previous use as a norm-referenced measure to assess performance relative to other test takers is no longer possible for any Step 1 examinations taken during or after January 2022. Today, Step 2 CK largely remains a norm-referenced exam providing residency programs standardized numerical values (1-300) to screen and compare applicants [10]. Prior to January 2022, Step 1 outcomes had a tremendous influence on the recruitment and selection of residents, acting as a rate-limiting step in reaching a new career milestone of being accepted into a desired residency ranging in different levels of competitiveness [11]. It has been reported that 94% of all National Residency Matching Program (NRMP) participating residency programs reported Step 1 as an important factor in selecting medical students to interview, with 68% of programs requiring a minimum target score [12].

The USMLE exams have shaped the culture of how students approach and value their medical education due to the emphasis placed on these two exams by the residency programs. The strategic preparation, planning, and study of a student are equivalent to, if not surpass, the emphasis that some residency programs previously placed on Step 1 as a measure of potential success. The vast majority of all medical students take a dedicated study period for both Step 1 and Step 2 exams at which time they study unmeasurable hours’ worth of material specific to the examinations [13]. It is not uncommon for students to experience imposter syndrome with their USMLE preparation and performance [14]. Together with the inherent stress of this process, it has negatively impacted the mental health and well-being of medical students across the globe [15]. Sharing many parallels to the evolutionary changes shaping medical education, the National Board of Medical Examiners (NBME) and Federation of State Medical Boards (FSMB) decision to convert Step 1 to a pass/fail score structure marked a historical change that will impose incredible influence on the next milestone of major changes that will shape medical education, career advising, and professional development [16].

The objective of this systematic review is to report on the literature that measures USMLE Step 1 and Step 2 CK as predictors of quality resident performance across all medical specialties and to better understand the ability of these exams to predict quality resident performance based on the available literature.

## Method

This systematic review was designed according to the Preferred Reporting Items for Systematic Reviews and Meta-Analyses (PRISMA) [17]. The guiding principle of this search strategy was to capture all studies related to the assessment of Step 1 or Step 2 CK as predictors/indicators of resident physician performance.

This review adapted a search strategy that investigated three databases. The original search strategy surveyed MEDLINE and was adapted to survey Cochrane Library and Embase. The framework for this search included the original language of publication and included original studies. The study period for inclusion was any article published after January 1990, as to include any article after the period during which the USMLE was established. Editorials and reviews were excluded. Key search terms were identified based on synonyms of the study basis (Step 1 and Step 2 CK) and study population (Residents of any ACGME-accredited specialty). The original, complete MEDLINE search strategy adapted for use on Cochrane Library and Embase is outlined in **Appendix E1**.

**Fig. 1:**
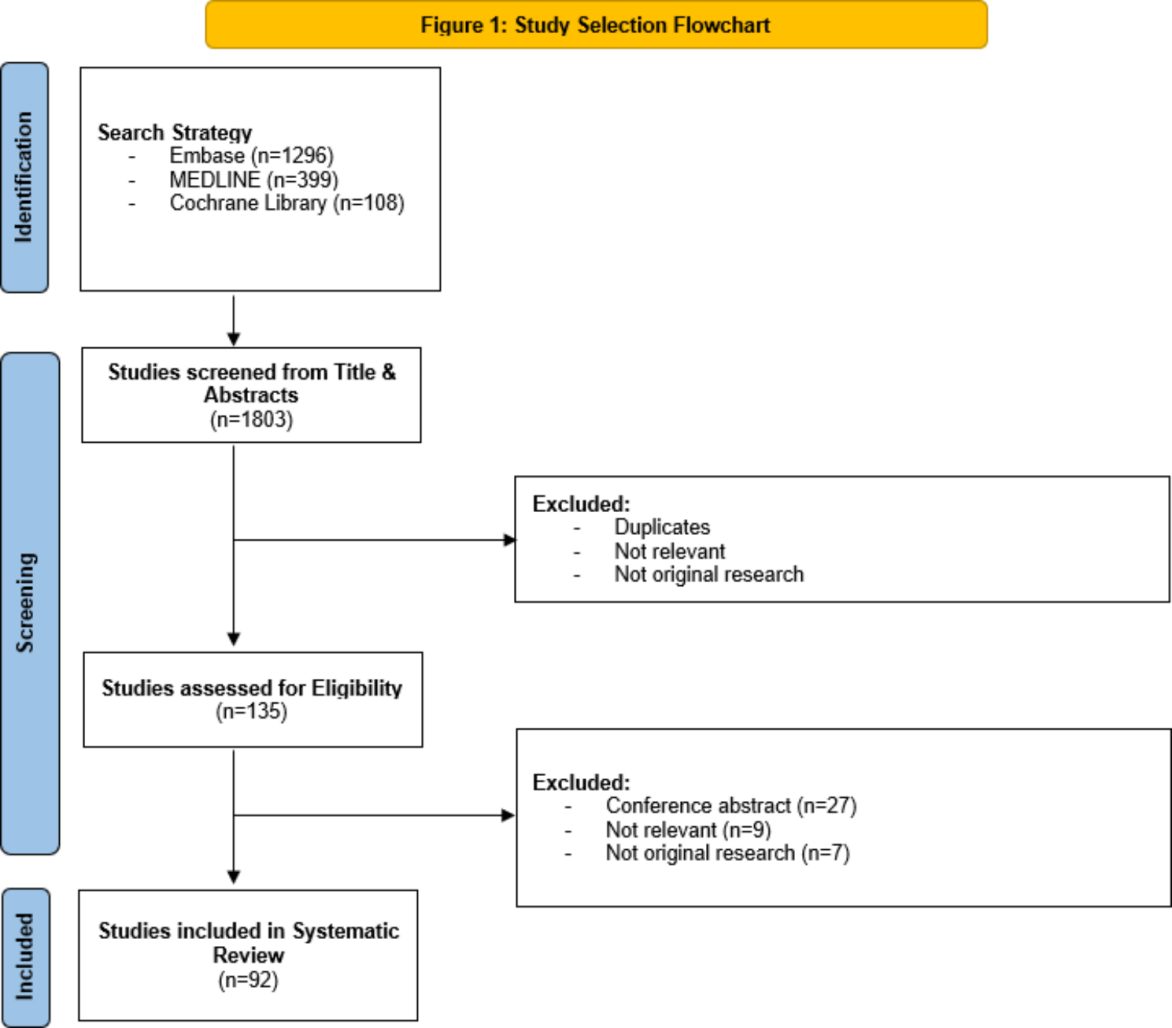
Study selection flowchart as designed according to the Preferred Reporting Items for Systematic Reviews and Meta-Analyses (PRISMA)

**Fig. 2:**
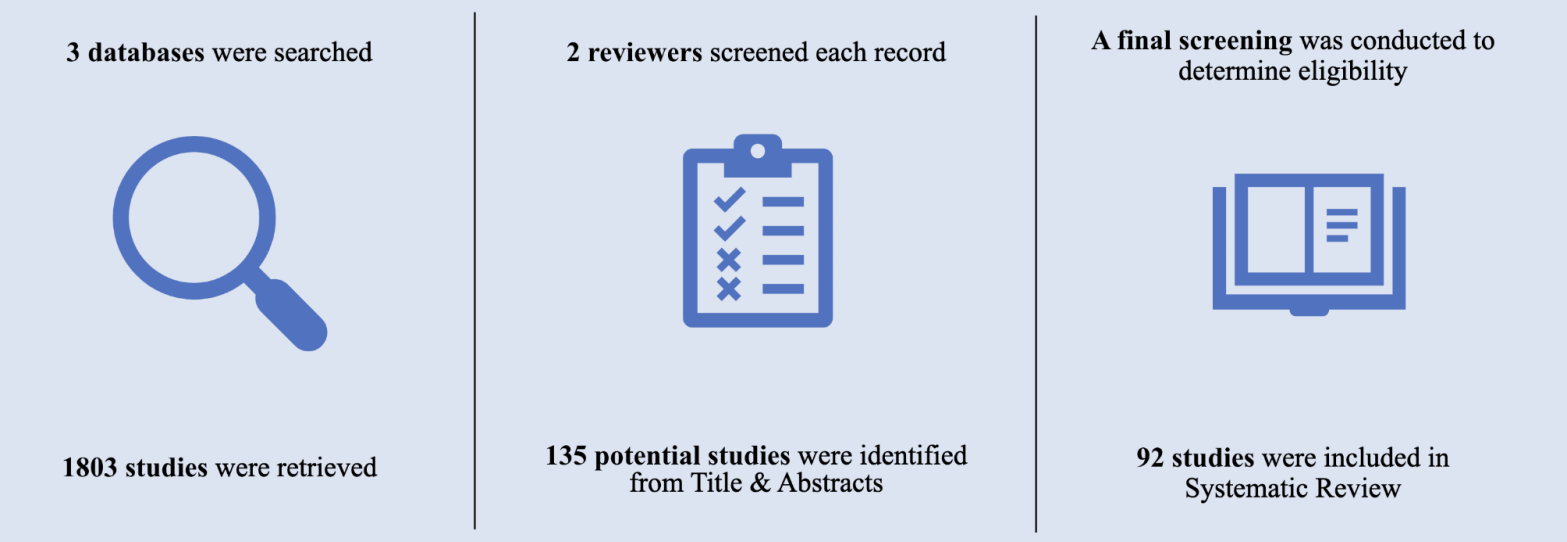
Summary figure outlining results of systematic review as designed according to the Preferred Reporting Items for Systematic Reviews and Meta-Analyses (PRISMA)

After completion of the search, irrelevant studies were excluded and duplicates were removed. The titles and abstracts of the remaining studies were individually screened by two authors, BR and NC, to determine eligibility. A study was deemed eligible if it provided all three of the following information: (a) Step 1 or Step 2 CK as indicators for (b) resident outcomes in (c) any ACGME-accredited specialty training program. Resident outcomes were considered any objective or subjective measures (examinations, evaluations, surveys, etc.) of resident performance. Both authors compared individually determining initial eligibility based on titles and abstracts and discussed any disagreements in-depth before forming a conclusion. Once the initial eligibility check was complete, full texts of each eligible article were examined before the final determination of inclusion was agreed upon by both screening authors. Using the Cochrane risk-of-bias tool, a risk-of-bias assessment was conducted on each article before inclusion to ensure a valid study design [18]. The complete search method can be appreciated in Figs. 1 and 2. Data collected from included studies were synthesized and presented in a narrative format, including a summary of the studies (Tables 1 and 2).

**Table 1:** USMLE Step 1 and/or 2 CK Correlation with Residency Performance; Stratified by Specialty

No.	Title	Authors	Year	Performance measure(s)	Data/Results
**ANESTHESIOLOGY**					
1	Anesthesiology Resident Performance on the US Medical Licensing Examination Predicts Success on the American Board of Anesthesiology BASIC Staged Examination: An Observational Study	Markham et al	2020	American Board of Anesthesiology (ABA) BASIC examination	**Step 1**: Independently predicted success on the ABA BASIC examination (OR 1.11, 95% CI 1.05–1.17, p < 0.001).
					**Step 2**: N/A.
2	Medical School Clinical Knowledge Exam Scores, Not Demographic or Other Factors, Associated With Residency In-Training Exam Performance	Patzkowski et al	2021	Anesthesiology in-training exam from third clinical year (CA-3 ITE)	**Step 1**: N/A.
					**Step 2**: Step 2 CK scores were significantly associated with CA-3 ITE scores (pooled β = 0.59, SE ± 0.12, p < 0.001).
					For every 1-point increase in Step 2 CK z-score, the CA-3 ITE z-score increased by 0.59 points.
3	The Utility of Pre-Residency Standardized Tests for Anesthesiology Resident Selection: The Place of United States Medical Licensing Examination Scores	Guffey et al	2011	Anesthesia Knowledge Test (AKT) ranking, PGY-1 IM ITE, CA-1 ITE, CA-2 ITE, ABA written board examination part 1	**Step 1 and Step 2**: Averaged Step 1 and Step 2 CK score, as well as each score individually, significantly correlated to all performance measures (p < 0.01).
					Averaged Step 1 and Step 2 CK examinations correlated to ABA written examination score (slope = 0.72, r = 0.48, p = 0.001).
**DERMATOLOGY**					
4	Correlation of USMLE Step 1 scores with performance on dermatology in-training examinations	Fening et al	2009	Dermatology ITE-1, ITE-2, ITE-3	**Step 1**: Step 1 scores had a positive, significant, moderate correlation with ITE scores for each year of residency (r = 0.467, 0.541, 0.527 for ITE-1, ITE-2, ITE-3 respectively, p < 0.001).
					Step 1 scores explained ~ 26% of ITE score variability, indicating other factors are involved.
					**Step 2**: N/A.
**EMERGENCY MEDICINE**					
5	A Critical Disconnect: Residency Selection Factors Lack Correlation With Intern Performance	Burkhardt et al	2020	Milestone level achieved in each of the 23 Emergency Medicine (EM) core competencies after PGY-1	**Step 1 and Step 2**: Neither Step 1 nor Step 2 CK scores were significantly associated with residents’ performance on the core competencies.
					**Step 2**: A statistically significant, yet minimal, very weak positive correlation was noted between Step 2 CK score and Medical Knowledge (MK) competency (r = 0.01, 95% CI 0-0.02).
6	Assessing Clinical Reasoning Skills in Scenarios of Uncertainty: Convergent Validity for a Script Concordance Test in an Emergency Medicine Clerkship and Residency	Humbert et al	2011	Emergency Medicine Script Concordance Test (EM-SCT)	**Step 1**: N/A.
					**Step 2**: Among M4 students who matched into EM residency programs, EM-SCT and Step 2 CK were significantly correlated (r = 0.56, p < 0.001).
7	Conference Attendance Does Not Correlate With Emergency Medicine Residency In-Training Examination Scores	Hern Jr. et al	2009	American Board of Emergency Medicine (ABEM) ITE	**Step 1**: Step 1 score was a predictor of ABEM-ITE score (coefficient = 0.186, 95% CI = 0.155–0.217; p < 0.001).
					**Step 2**: N/A.
8	Correlation of the National Board of Medical Examiners Emergency Medicine Advanced Clinical Examination Given in July to Intern American Board of Emergency Medicine in-training Examination Scores: A Predictor of Performance?	Hiller et al	2015	Emergency Medicine Advanced Clinical Examination (EM-ACE), ABEM ITE-1	**Step 1 and Step 2**: In a linear regression model utilizing every available variable, neither Step 1 (p = 0.61) nor Step 2 CK (p = 0.53) score was associated with ABEM ITE-1 score.
					However, significant collinearity was observed among the EM-ACE, ABEM ITE-1, and both Step scores (r ranged from 0.58–0.70).
9	Do United States Medical Licensing Examination (USMLE) Scores Predict In-Training Test Performance for Emergency Medicine Residents?	Thundiyil et al	2008	ABEM ITE-1, ITE-2, ITE-3	**Step 1**: There was a mild correlation between Step 1 scores and ABEM ITEs (R-squared values of 0.25, 0.18, 0.16 for ABEM ITE-1, ITE-2, ITE-3, respectively).
					**Step 2**: There was a moderate correlation between Step 2 CK scores and ABEM ITEs (R-squared values of 0.43, 0.44, 0.38 for ABEM ITE-1, ITE-2, ITE-3, respectively).
					**Step 1 and Step 2**: Step 1 or Step 2 CK scores below 200 were associated with significantly poorer performance on ABEM ITEs compared to scores above 200 (p < 0.05).
10	Does the National Resident Match Program Rank List Predict Success in Emergency Medicine Residency Programs?	Meter et al	2016	Resident Graduation Rank (GR) as determined by ACGME core competencies	**Step 1**: Only a weak, statistically insignificant positive correlation was found between Step 1 score ranking among residents and GR (r = 0.097, p = 0.122).
					**Step 2**: N/A.
11	Emergency Medicine Residency Applicant Characteristics Associated with Measured Adverse Outcomes During Residency	Bohrer- Clancy et al	2018	Measured negative resident outcomes, including: letters of reprimand (LoR), letters of deficiency (LoD), extension of residency (EXT), and failure to finish residency (DNF)	**Step 1**: Residents who had failed Step 1 during medical school were significantly more likely to experience a negative outcome during training (46.2% vs. 17.5%, p = 0.020).
					**Step 2**: N/A.
12	Exploring the Association Between USMLE Scores and ACGME Milestone Ratings: A Validity Study Using National Data From Emergency Medicine	Hamstra et al	2021	ACGME EM Milestone ratings for selected subcompetencies: MK-01, Patient Care (PC) 04, PC-05, PC-06, PC-08, PC-09, Professionalism (PR) 01, Systems-based practice (SBP) 02, Interpersonal and Communication skills (ICS) 01	**Step 1**: Step 1 score showed a small but significant effect with only the MK-01 subcompetency (r = 0.06 [95% CI, 0.05–0.07], p < .05).
					**Step 2**: A small but statistically significant positive relationship was found between Step 2 CK score and each of the selected subcompetencies (r ranged from 0.02 [95% confidence interval (CI), 0.01–0.03] to 0.12 [95% CI, 0.11–0.13]; all p < .05).
					PR-01 and ICS-01 were the subcompetencies with the lowest correlations with Step 2 CK score.
13	Predicting American Board of Emergency Medicine Qualifying Examination Passage Using United States Medical Licensing Examination Step Scores	Caffery et al	2018	First-attempt passage of the ABEM qualifying (written) examination	**Step 1**: In a multivariable logistic regression model, Step 1 was not a predictor of ABEM qualifying examination passage (OR = 1.02 [95% CI 0.99–1.05, p < 0.17]).
					**Step 2**: In a multivariable logistic regression model, Step 2 CK scores could predict the odds of passing the ABEM qualifying examination (OR = 1.05 [95% CI 1.02–1.08, p < 0.001]).
14	Predictors of a Top Performer During Emergency Medicine Residency	Bhat et al	2015	A resident’s placement in the top third of his/her graduating class, based on performance on final ACGME core competency semi-annual evaluation	**Step 1**: Step 1 score was a weak but statistically significant predictor of top performers in EM residency (OR = 1.02 [95% CI 1.01–1.04, p = 0.004]).
					**Step 2**: Step 2 CK score was not a statistically significant predictor of top performers in EM residency (OR = 1.01 [95% CI 1.00–1.03, p = 0.100]).
15	USMLE Scores Predict Success in ABEM Initial Certification: A Multicenter Study	Harmouche et al	2017	First-attempt ABEM qualifying (written) examination, First-attempt ABEM oral certification examination	**Step 1 and Step 2**: Residents who passed written boards had significantly higher USMLE Step 1, Step 2 CK, and composite (sum of Step 1 and 2 CK) scores (p < .001).
					**Step 2**: Residents who passed both boards had significantly higher Step 2 CK and composite scores (p < .001).
16	What Predicts Performance? A Multicenter Study Examining the Association Between Resident Performance, Rank List Position, and United States Medical Licensing Examination Step 1 Scores	Wagner et al	2016	Resident Graduation Rank Order List (ROL), as determined by anonymous surveys filled by full-time EM faculty	**Step 1**: The rank of Step 1 score within each class had no correlation with Graduation ROL (Rho = 0.15, p = 0.14).
					**Step 2**: N/A.
**FAMILY MEDICINE**					
17	Associations Between Medical Education Assessments and American Board of Family Medicine Certification Examination Score and Failure to Obtain Certification	Peterson et al	2020	American Board of Family Medicine (ABFM) certification examination, ABFM certification status	**Step 1**: N/A.
					**Step 2**: Step 2 CK was predictive of ABFM certification examination scores and ABFM certification status in all statistical models (p < 0.05).
18	One Institution’s evaluation of family medicine residency applicant data for academic predictors of success	Busha et al	2021	PGY-1 ABFM ITE, six ACGME competency domains	**Step 1**: There was a moderate and statistically significant association between PGY-1 ABFM ITE and Step 1 score (r = 0.466, p < 0.001).
					**Step 2**: There was a moderate and statistically significant association between PGY-1 ABFM ITE and Step 2 CK score (r = 0.475, p < 0.001).
					**Step 1 and Step 2**: Neither Step 1 nor Step 2 CK had a correlation with resident assessment across any of the ACGME competency domains.
**FELLOWSHIP - HEMATOLOGY AND MEDICAL ONCOLOGY**					
19	Identification of Factors Associated with Hematology-Oncology Fellow Academic Success and Career Choice	Marshall et al	2018	Hematology (H) ITE-1, ITE-2, ITE-3, Oncology (O) ITE-1, ITE-2, ITE-3, Awards during fellowship, Abstracts during fellowship, Publications during fellowship	**Step 1**: Higher Step 1 scores were significantly associated with higher H ITE-1, H ITE-2, H ITE-3, and O ITE-2 scores (all p < 0.05).
					**Step 2**: Higher Step 2 CK scores were significantly associated with higher H ITE-1, H ITE-2, H ITE-3, O ITE-1, and O ITE-2 scores (all p < 0.05).
					**Step 1 and Step 2**: Neither Step 1 nor Step 2 CK was associated with number of Awards, Abstracts, or Publications during fellowship.
20	Medical Knowledge Assessment by Hematology and Medical Oncology In-Training Examinations Are Better Than Program Director Assessments at Predicting Subspecialty Certification Examination Performance	Collichio et al	2016	ABIM Hematology certification examination, ABIM Medical Oncology certification examination	**Step 1**: Step 1 score was a weak, yet statistically significant, predictor of ABIM Hematology certification examination scores (Standardized Coefficient (*β*) = 0.14, p < 0.0001).
					Step 1 score was a weak, yet statistically significant, predictor of ABIM Medical Oncology certification examination scores (Standardized Coefficient (*β*) = 0.14, p < 0.0001).
					**Step 2**: N/A.
**FELLOWSHIP - INFECTIOUS DISEASE**					
21	The Relationship Between Performance on the Infectious Diseases In-Training and Certification Examinations	Grabovsky et al	2014	ABIM Infectious Disease certification examination score, ABIM Infectious Disease certification examination passing status	**Step 1**: Step 1 score was a statistically significant predictor of ABIM Infectious Disease certification examination score (β = 0.202, p < 0.001).
					**Step 2**: Step 2 CK was a statistically significant predictor of ABIM Infectious Disease certification examination passing status (OR = 1.036, p < 0.001).
**FELLOWSHIP - NEURORADIOLOGY**					
22	United States Medical Licensing Examination Step 1 and 2 Scores Predict Neuroradiology Fellowship Success	Yousem et al	2016	Fellow E*Value scores as determined by faculty assessment of ACGME core competencies, Best-to-worst ranking within fellowship cohort	**Step 1**: The predicted probability of being ranked in top three was 63.4% if Step 1 score was 260 and 24.1% if Step 1 score was 200.
					The mean Step 1 score of top-ranked fellows (234.5) was significantly higher than that of bottom-ranked fellows (217.7).
					**Step 1 and Step 2**: Fellowship E*Value scores and rankings showed statistically significant correlations with all USMLE scores, especially Step 1 (p < 0.05).
**INTERNAL MEDICINE**					
23	Association Between Internal Medicine Residency Applicant Characteristics and Performance on ACGME Milestones During Intern Year	Golden et al	2021	Mean Milestone performance across all 22 Internal Medicine (IM) ACGME subcompetencies	**Step 1 and Step 2**: Neither Step 1 nor Step 2 CK were associated with Milestone performance.
24	Associations Between United States Medical Licensing Examination (USMLE) and Internal Medicine In-Training Examination (IM-ITE) Scores	McDonald et al	2008	IM-ITE	**Step 1 and Step 2**: USMLE scores explained 17%-27% of the variance in IM-ITE scores.
					Regression coefficients (95% CI) for adjusted associations with IM-ITE scores were Step 1: 0.19 (0.12–0.27), Step 2 CK: 0.23 (0.17–0.30).
25	Correlation between housestaff performance on the United States Medical Licensing Examination and standardized patient encounters	Rifkin and Rifkin	2005	Structured clinical exam performance using standardized patients	**Step 1 and Step 2**: Neither Step 1 nor Step 2 CK correlated with structured clinical exam performance (step 1 r = 0.2, df = 32, p = 0.27; step 2 r = 0.09, df = 30, p = 0.61)
26	Correlation of United States Medical Licensing Examination and Internal Medicine In-Training Examination performance	Perez Jr. and Greer	2009	IM ITE-1, IM ITE-2, IM ITE-3	**Step 1**: r values for Step 1 and ITE percent correct in PGY I, II and III were 0.46, 0.55 and 0.51, respectively (p < 0.05).
					**Step 2**: r values for Step 2 CK and ITE percent correct in PGY I, II and III were 0.79, 0.70 and 0.72, respectively (p < 0.05).
27	Correlations Between the USMLE Step Examinations, American College of Physicians In-Training Examination, and ABIM Internal Medicine Certification Examination	McDonald et al	2020	ABIM Certification Examination (ABIM-CE)	**Step 1**: Step 1 was a predictor of ABIM-CE scores [Rescaled b coefficient at 0.5 SD (95% CI): 4.63 (3.81–5.44), p < 0.001].
					**Step 2**: Step 2 CK showed a slightly stronger association with ABIM-CE scores [Rescaled b coefficient at 0.5 SD (95% CI): 5.95 (5.02–6.88), p < 0.001].
28	Developing a Predictive Model to Assess Applicants to an Internal Medicine Residency	Neely et al	2010	Overall 3-year performance rating as determined by clinical reasoning and knowledge, leadership, professionalism, patient care, and teaching	**Step 1**: A 10-point-lower Step 1 score correlated with an additional 2% increase (worse) resident performance rating (p = 0.01)
					*note: scoring system had “lower” values indicating “stronger” performance.
					**Step 2**: N/A.
29	Do the selection criteria of internal medicine residency program predict resident performance?	Rahil et al	2021	IM-ITE 1–4, cumulative score of formative evaluation based on ACGME core competencies, Arab Board (part 1 and 2) Written exam, Arab Board Clinical Exam	**Step 1**: N/A.
					**Step 2**: ITE score correlated positively with Step 2 CK score (r = 0.621, r = 0.587, r = 0.576, r = 0.571, p, 0.001) for ITE-1, ITE-2, ITE-3, and ITE-4, respectively.
					Step 2 CK score was not associated with any other performance measure.
30	Do USMLE steps, and ITE score predict the American Board of Internal Medicine Certifying Exam results?	Rayamajhi et al	2020	ABIM-CE	**Step 1 and Step 2**: 100% of residents who failed ABIM-CE had Step 1 scores < 220, and 20% of residents who failed ABIM-CE had Step 2 CK scores < 220.
					The probability of passing ABIM-CE was ~ 80% with USMLE scores > 200, and almost 100% with USMLE scores > 250.
					There was a significant correlation of passing ABIM-CE with 10 points increase in Step 1 (OR = 2.70; 95% CI: 1.38–5.29) and 10 points increase in Step 2 CK (OR = 2.31; 95% CI 1.33–4.01).
31	The Relationship Between Internal Medicine Residency Graduate Performance on the ABIM Certifying Examination, Yearly In-Service Training Examinations, and the USMLE Step 1 Examination	Kay et al	2015	ABIM-CE	**Step 1**: Step 1 scores and ABIM-CE scores had a modest positive correlation (rho: 0.59).
					Failing Step 1 previously significantly increased the likelihood of failing ABIM-CE (RR: 7.1; 95% CI: 3.7–13.2).
					**Step 2**: N/A.
32	USMLE Step 2 CK: Best Predictor of Multimodal Performance in an Internal Medicine Residency	Sharma et al	2019	Long block 360-degree ratings as determined by patient care, teamwork, professionalism, and efficiency	**Step 1**: In a multivariable analysis, Step 1 scores were associated with IM-ITE scores (ITE-1: r = 0.240, p = 0.020; ITE-2: r = 0.271, p = 0.008; ITE-3: r = 0.332, p = 0.023).
				Direct patient assessment of residents on physician attributes: explains, listens, gives instructions, knows history, respects patient, is on time, calls with results	Step 1 score was not associated with any other performance measure in the multivariable analysis.
				IM-ITE scores	**Step 2**: In a multivariable analysis, Step 2 CK scores were the most predictive across all residency performance measures (all p < 0.05).
				ABIM-CE status on first attempt	
**NEUROLOGICAL SURGERY**					
33	United States Medical Licensing Examination Step 1 Scores Directly Correlate with American Board of Neurological Surgery Scores: A Single-Institution Experience	Nagasawa et al	2016	ABNS (American Board of Neurological Surgery) scores	**Step 1**: USMLE Step 1 scores were found to be significantly correlated with ABNS scores (P = 0.01, Spearman correlation coefficient of 0.7).
					**Step 2**: N/A.
34	United States Medical Licensing Examination step 2 scores do not predict American Board of Neurological Surgery scores: A single-institution experience	Udawatta et al	2019	ABNS (American Board of Neurological Surgery) scores	**Step 1**: N/A.
					**Step 2**: USMLE Step 2 ABNS scores were not found to be significantly correlated (Pearson Correlation of 0.228 with a 2-tailed significance of 0.272).
**OBSTETRICS AND GYNECOLOGY**					
35	Board certification in obstetrics and gynecology: associations with physicians’ demographics and performances during medical school	Silber et al	2004	Board Certification	**Step 1**: No significant association.
					**Step 2**: Logistic regression indicated that scores on Step 2 CK were the most important predictor of achieving board certification. A score of 200 or higher on Step 2 CK was 7 times more likely to result in board certification versus a score below 200 (Only Step 2 had a statistically significant (P < .03) relationship with board certification. The odds ratio, dichotomized at the mean score of 200 (for Step 2), was 7.1.
36	Do U.S. Medical Licensure Examination Step 1 scores correlate with Council on Resident Education in Obstetrics and Gynecology in-training examination scores and American Board of Obstetrics and Gynecology written examination performance?	Armstrong et al	2007	Council on Resident Education in Obstetrics and Gynecology (CREOG) in-training examinations and the pass rate on the American Board of Obstetrics and Gynecology (ABOG) written examination	**Step 1**: USMLE step 1 scores were correlated with CREOG in-training examination scores (p < 0.000).
					**Step 2**: N/A.
**OPHTHALMOLOGY**					
37	A comparative study of resident performance on standardized training examinations and the American board of ophthalmology written examination	Johnson et al	2010	Ophthalmic Knowledge Assessment Program (OKAP) exam and the American Board of Ophthalmology written qualifying examination (ABO-WQE)	**Step 1**: Step 1 was not significantly associated with ABO-WQE performance. It should be noted that the ABO-WQE pass rate, however, was significantly associated with OKAP examination scores during the 3 residency years.
					Year 1: odds ratio [OR], 8.85 and 95% confidence interval [CI] 1.82–42.79;
					Year 2: OR, 5.28 and 95% CI, 1.15–25.27;
					Year 3: OR, 11.08 and 95% CI, 1.86–68.96.
					**Step 2**: N/A.
38	A multicenter analysis of the ophthalmic knowledge assessment program and American board of ophthalmology written qualifying examination performance	Lee et al	2012	American Board of Ophthalmology Written Qualifying Examination (WQE)	**Step 1**: The mean score on Step 1 and all 3 OKAP examinations of those that passed the WQE was significantly higher compared with that of those who failed.
					However, USMLE score had the smallest area under the
					ROC curve (0.70) (weakest predictor) out of the compared predictors.
					**Step 2**: N/A.
39	Resident and program characteristics that impact performance on the Ophthalmic Knowledge Assessment Program (OKAP)	Zafar et al	2019	Ophthalmic Knowledge Assessment Program (OKAP) performance	**Step 1**: Higher step scores were found to be predictive of residents scoring ≥ 75th percentile on the OKAP (OR = 2.48, [95% CI: 1.68–3.64, p < 0.001]).
					**Step 2**: N/A.
**ORTHOPEDIC SURGERY**					
40	Do scores of the USMLE step 1 and OITE correlate with the ABOS part I certifying examination?: A multicenter study	Dougherty et al	2009	ABOS Part I certifying examination	**Step 1**: Step 1 scores showed a correlation [correlation coefficient of 0.53] with ABOS examination scores.
					**Step 2**: N/A.
41	Does residency selection criteria predict performance in orthopaedic surgery residency?	Raman et al	2015	American Board of Orthopaedic Surgery (ABOS) Part I scores, Orthopaedics In-Training Exam (OITE) scores, subjective ratings by faculty including global evaluation scores and faculty rankings of residents	**Step 1**: N/A.
					**Step 2**: ABOS scores had a moderate linear correlation with the Step 2 CK (r = 0.55, p < 0.001). It should be noted for context that the number of clinical honors received also showed a correlation with ABOS scores (r = 0.45, p < 0.001).
					OITE scores had a weak linear correlation with both number of clinical honors (r = 0.35, p = 0.009) and Step 2 CK scores (r = 0.29, p = 0.02).
42	Factors Predictive of Orthopaedic In-training Examination Performance and Research Productivity Among Orthopaedic Residents	Kreitz et al	2018	OITE performance	**Step 1**: A significant positive correlation was found between the USMLE step 1 score and the most recent OITE performance percentile (P < 0.001).
					**Step 2**: Also found was a significant positive correlation between the USMLE step 2 CK score and OITE performance (P < 0.001).
43	Orthopaedic in-training examination scores: A correlation with USMLE results	Black et al	2006	Orthopaedic In-Training Examination scores	**Step 1**: No association found.
					**Step 2**: Significant moderate-sized correlation was found between United States Medical Licensing Examination Step 2 scores and Orthopaedic In-Training Examination score percentiles (p < 0.05).
44	Predictive measures of a resident’s performance on written Orthopaedic Board scores	Drystad et al	2011	Written boards; OITEs	**Step 1**: Positive correlation with written boards (r^2^ = 0.32, p = 0.0011).
					**Step 2**: Positive correlation (r^2^ = 0.42, p = 0.0014).
					Step 2 correlates with OITE scores.
45	Relationship among United States Medical Licensing Step I, orthopedic in-training, subjective clinical performance evaluations, and american board of orthopedic surgery examination scores: a 12-year review of an orthopedic surgery residency program	Crawford et al	2010	OITE YIT percentile rank score	**Step 1**: No significant relationship was found between OITE and USMLE Step 1 (3-digit) score (r = 0.22, p = 0.13).
					**Step 2**: N/A.
46	Relationship between performance on part I of the American board of orthopaedic surgery certifying examination and scores on USMLE Steps 1 and 2	Swanson et al	2009	American Board of Orthopaedic Surgery (ABOS) Certifying Examination	**Step 1**: moderately strong correlation found (P < 0.01, r = 0.527).
					**Step 2**: moderately strong correlation found (P < 0.01, r = 0.531).
47	What predicts outstanding orthopedic residents among the program?	Claessen et al	2019	OITE score	**Step 1**: USMLE Step 1 score (P = 0.0038) was directly associated with higher OITE score.
					**Step 2**: N/A.
**OTHER**					
48	Comparative values of medical school assessments in the prediction of internship performance	Lee et al	2018	The outcome measure was the internship performance ratings of graduates provided by residency program directors who participated in an annual survey from 2012 to 2015 conducted by the school. The ratings were based on a 3-point Likert-type scale assessing the same nine areas as those included in the clerkship evaluation	**Step 1**: No association found.
					**Step 2**: Step 2 CK was found to be correlated with internship performance (Beta = 0.14, t = 2.09, p < 0.05).
					[Wide spectrum of specialty areas, with 32% in IM and about 10% each in family medicine, emergency medicine, surgery, and pediatrics]
49	Identifying medical students likely to exhibit poor professionalism and knowledge during internship	Greenburg et al	2007	There was a total of 1,559 graduates from USU. Intern program director surveys were collected from 1,247 (80%)	**Step 1**: USMLE Step 1 scores (OR = 1.03; 95% CI = 1.01–1.05) were directly correlated with knowledge based aspects of resident performance, but had no association with professionalism.
					**Step 2**: N/A.
50	Development and initial validation of a program director’s evaluation form for third-year residents	Artino et al	2015	PGY-3 PD ratings (“Medical Expertise” and “Professionalism” scores on a survey)	**Step 1**: No association found (r ~ 0).
					**Step 2**: No association found (r ~ 0).
					…
					[The breakdown of program director response proportions: family medicine (n = 84, 22%), internal medicine (n = 49, 13%), pediatrics (n = 47, 12%), surgery (n = 39, 10%), psychiatry (n = 25, 6%), emergency medicine (n = 18, 5%), and radiology (n = 15, 4%)
					Step 1 and Step 2 CK have weak r values close to zero with relation to resident medical expertise and professionalism]
51	Are commonly used premedical school or medical school measures associated with board certification?	Durning et al	2015	Achieving board certification in an American Board of Medical Specialties specialty (many specialties)	**Step 1**: Positive correlation found (r = 0.066).
					**Step 2**: Positive correlation found (r = 0.040).
52	Relationship Between Standardized Test Scores and Board Certification Exams in a Combined Internal Medicine/Pediatrics Residency Program	Ost et al	2021	In-Training Exams in Internal Medicine (ITE-IM) and Pediatrics (ITE-P) were analyzed with the corresponding ABIM-CE and ABP-CE first-time scores	**Step 1**: Positive correlation found (r^2^ = 0.35, p < .001).
					**Step 2**: Positive correlation found (r^2^ = 0.30, p < .001).
					[In one Med/Peds program, USMLE Step 1 and 2 and all ITE-IM and ITE-P scores predicted certifying exam scores and passage]
53	The feasibility, reliability, and validity of a program director’s (supervisor’s) evaluation form for medical school graduates	Durning	2005	Evaluation form with a 6 point scale. Covers medical expertise and professionalism	**Step 1**: Positive correlation found with expertise (pearson coefficient = 0.26; p value < .0001).
					**Step 2**: Positive correlation found with expertise (pearson coefficient = 0.32; p value < .0001).
					[USMLE Step 1 and 2 scores correlated with expertise but not with professionalism]
54	The impact of postgraduate training on USMLE Step 3 and its computer-based case simulation component	Feinberg et al	2011	CCS portion of Step 3	**Step 1**: Positive correlation found.
					**Step 2**: Positive correlation found.
					Predictors of Step 1 and Step 2 CK explained 55% of overall Step 3 variability and 9% of CCS score variability.
55	Objective predictors of intern performance	Filiberto et al	2021	PDs provided a global assessment rating and ratings	**Step 1**: Positive correlation found (p = 0.006).
				addressing ACGME competencies (response rate = 47%) with five response options: excellent = 5, very good = 4,	
				acceptable = 3, marginal = 2, unacceptable = 1. PDs also classified interns as outstanding = 4, above average = 3,	**Step 2**: Positive correlation found (p = 0.030).
				average = 2, and below average = 1	
					Better performance as an intern was associated with higher USMLE scores.
56	Predicting Performance of First-Year Residents: Correlations Between Structured Interview, Licensure Exam, and Competency Scores in a Multi-Institutional Study	Marcus-Blank et al	2015	Year-end overall performance and year-end performance on patient care, interpersonal and communication skills	**Step 1**: Positive correlation found (r = .18, P < .05).
					**Step 2**: Positive correlation found (r = .19, P < 0.05).
					Structured interview questions are helpful in predicting residency performance. USMLE
					scores contributed incremental validity over SI scores in predicting year-end performance
					overall and on patient care and medical knowledge.
57	Factors associated with american board of medical specialties member board certification among US Medical School Graduates	Jeffe et al	2011	ABMS member board certification (many specialties)	**Step 1**: N/A.
					**Step 2**: Positive correlation found.
					Graduates in all specialty categories with first-attempt passing scores in the highest tertile (vs first-attempt failing scores) on US Medical Licensing Examination Step 2 CK were more likely to be board certified.
58	Relative United States Medical Licensing Examination (USMLE) Performance by Specialty Is Not a Predictor of Board Exam Pass Rate: The Case of Diagnostic Radiology	Sakya et al	2021	Boards passage rates for radiology (other specialty data also mentioned)	**Step 1**: No statistically significant correlation found between Step 1 and board passage rates; Spearman rho coefficient of 0.0679 (p = 0.8101).
					**Step 2**: No statistically significant correlation found between Step 2 CK and board passage rates; Spearman rho coefficient of 0.1430 (p = 0.6257).
**OTOLARYNGOLOGY - HEAD AND NECK SURGERY**					
59	The impact of a resident-run review curriculum and USMLE scores on the Otolaryngology in-service exam	Redmann et al	2017	OTE scores	**Step 1**: There was a moderate correlation found between USMLE Step 1 scores and OTE scores (r = .33 to .65 for all PG years).
					**Step 2**: There was a moderate correlation found between USMLE Step 2 CK scores and OTE scores (r = .39 to .55 for all PG years).
60	USMLE and Otolaryngology: Predicting Board Performance	Puscas et al	2017	WQE passage	**Step 1**: positive correlation; odds ratio 1.07, p-value < 0.001.
					**Step 2**: positive correlation; odds ratio 1.04, p-value < 0.001.
**PATHOLOGY**					
61	United states medical licensing examination step 1 two-digit score: A correlation with the american board of pathology first-time test taker pass/fail rate at the university of Pittsburgh medical center	Picarsic et al	2011	ABP passage / failure	**Step 1**: USMLE step 1 scores are correlated with ABP first-time pass/failure rates.
					**Step 2**: N/A.
**PEDIATRICS**					
62	Application Factors Associated With Clinical Performance During Pediatric Internship	Gross et al	2020	Accreditation Council for Graduate Medical Education pediatric Milestone scores	**Step 1**: No association between USMLE Step 1 performance and milestone scores were found.
					**Step 2**: N/A.
63	United States Medical Licensing Examination and American Board of Pediatrics Certification Examination Results: Does the Residency Program Contribute to Trainee Achievement	Welch et al	2017	American Board of Pediatrics Certification Examination	**Step 1**: Positive correlation found (b = .002, t = 2.54, P = .011).
					**Step 2**: Positive correlation found (b = .002, t = 2.54, P = .011).
					Linear regression analyses indicated that Step 2 results were a better predictor of ABP performance than Step 1 or a combination of the two USMLE scores.
64	USMLE Step 1 Scores as a Significant Predictor of Future Board Passage in Pediatrics	McCaskill et al	2007	American Board of Pediatrics (ABP) board certifying exam scores within a Pediatric residency-training program	**Step 1**: Only USMLE Step 1 scores (compared to Step 2) had a statistically significant association with board performance.
					**Step 2**: No association found.
**PSYCHIATRY**					
65	Selection Factors Among International Medical	Shiroma et al	2010	Psychiatry Resident-In-Training Examination (PRITE), Psychotherapy treatment session evaluations	**Step 1**: USMLE Step 1 was significantly correlated with PRITE outcomes (r = 0.37, P < 0.003).
	Graduates and Psychiatric Residency Performance				
					**Step 2**: USMLE Step 2 was significantly correlated with PRITE outcomes (r = 0.40; p < 0.003).
66	US Medical Licensing Exam Scores and Performance	Miller et al	2014	Psychiatry Resident-In-Training Examination (PRITE)	**Step 1**: Step 1 was significantly correlated with PRITE psychiatry and neurology scores (p < 0.01).
	on the Psychiatry Resident In-Training Examination				**Step 2**: Step 2 was significantly correlated with PRITE psychiatry and neurology scores (p < 0.01).
**RADIOLOGY**					
67	Do Residency Selection Factors Predict Radiology Resident Performance?	Agarwal et al	2017	Cumulative major discordance rate for on-call cases. Essentially rate of discrepancies between the preliminary resident case interpretation and ultimate attending interpretation that could impact patient care	**Step 1**: Higher USMLE Step 1 score predicted lower major discordance rates (p = 0.01).
					**Step 2**: N/A.
68	Does Medical School Performance Predict Radiology Resident Performance?	Boyse et al	2002	Rotation evaluations, retrospective faculty recall scores, ACR and ABR examination scores	**Step 1**: High USMLE Step 1 score predicted success on ABR written clinical examination, but did not predict rotation performance.
					**Step 2**: N/A.
69	Predictors for Failing the	Horn Jr. et al	2019	ABR core examination failure	**Step 1**: USMLE Step 1 performance significantly associated with ABR core examination failure (p = 0.041).
	American Board of Radiology Core Examination				**Step 2**: USMLE Step 2 performance significantly associated with ABR core examination failure (p = 0.043).
70	Predictors of Success on the ABR Core Examination	Calisi et al	2019	ABR core examination scores	**Step 1**: Higher Step 1 score significantly correlated with higher ABR core examination pass rate (p < 0.05).
					**Step 2**: Higher Step 2 score significantly correlated with higher ABR core examination pass rate (p < 0.05).
71	The Relationship Between US Medical Licensing Examination Step Scores and ABR Core Examination Outcome and Performance: A Multi-institutional Study	Patel et al	2020	ABR core examination scores	**Step 1**: Lower Step 1 tertile scores significantly correlated with lower core examination performance and higher core examination failure rates (P < 0.05).
					**Step 2**: Lower Step 2 tertile scores significantly correlated with lower core examination performance and higher core examination failure rates (P < 0.05).
**SURGERY**					
72	A Structured Educational Curriculum Including Online Training Positively Impacts American Board of Surgery In-Training	Kelly et al	2015	American Board of Surgery In-Training Examination (ABSITE) Scores	**Step 1**: USMLE Step 1 scores positively correlated with ABSITE scores (p < 0.001).
	Examination Scores				**Step 2**: N/A.
73	Achievement in Surgical Residency: Are Objective Measures of Performance Associated With Awards Received in Final Years of Training?	Mainthia et al	2014	Best Resident in Research award, Best Resident in Teaching award, Best Resident Overall award	**Step 1**: Award winners had significantly lower median Step 1 scores (p = 0.04).
					**Step 2**: Award winners had marginally lower Step 2 scores (p = 0.05).
74	American Board of Surgery examinations: can we identify surgery residency applicants and residents who will pass the examinations on the first attempt?	Shellito et al	2010	American Board of Surgery qualifying and certifying examination first-attempt pass rates	**Step 1**: Being among the top 50% of Step 1 scores was a significant objective predictor of first-attempt pass of ABS qualifying/certifying exams.
					**Step 2**: Being among the top 75% of Step 2 scores was a significant objective predictor of first-attempt pass of ABS qualifying/certifying exams.
75	Can We Predict Which Residents Are Going to Pass/Fail the Oral Boards?	Maker et al	2012	First-attempt pass rates on American Board of Surgery Certifying Examination (ABSCE)/oral boards	**Step 1**: No significant association.
					**Step 2**: ABSCE scores significantly correlated with Step 2 CK scores (p = 0.02).
76	Common attributes of high/low performing general surgery programs as they relate to QE/CE pass rates	Bankhead-Kendall et al	2016	American Board of Surgery qualifying and certifying examination first-attempt pass rates	**Step 1 and Step 2**: Surgery residency programs in the top 5% of ABS qualifying and certifying exams pass rates had significantly higher mean Step 1 and Step 2 CK scores.
77	Does Resident Ranking During Recruitment Accurately Predict Subsequent Performance as a Surgical Resident?	Fryer et al	2012	ABSITE Scores, PGY1 resident evaluation grade, overall evaluation grade, independent faculty ranking	**Step 1**: Step 1 scores were predictive of ABSITE scores only (P = 0.0057)
					**Step 2**: N/A.
78	Emotional Intelligence in Surgery is Associated with Resident Job Satisfaction	Hollis et al	2016	Emotional intelligence based on Trait EI Questionnaire (TEIQ)	**Step 1**: No significant association.
					**Step 2**: Step 2 CK score predictive of emotional intelligence (r = 0.46; p = 0.01).
79	Evaluation of Validity Evidence for Personality,	Gardner et al	2017	Faculty evaluations, professionalism, case logs, ABSITE, scholarly activity, Medical student evaluations of resident	**Step 1**: Step 1 score was associated with overall performance (p = 0.03).
	Emotional Intelligence, and Situational Judgment Tests to Identify Successful Residents				**Step 2**: No significant association.
80	Factors that Predict an Intern’s First ABSITE Score are Known by September	Aljamal et al	2018	ABSITE scores	**Step 1**: Step 1 was positively correlated with ABSITE scores (p < 0.05).
					**Step 2**: Step 2 was positively correlated with ABSITE scores (p < 0.05).
81	General Surgery Resident Remediation and Attrition	Yaghoubian et al	2012	Resident remediation and attrition rates	**Step 1**: Remediation was associated with Step 1 score (OR = 0.9, P = 0.02).
					**Step 2**: No significant association.
82	Is USMLE Step 1 score a valid predictor of success in surgical residency?	Sutton et al	2014	Rotation evaluations, ‘drop outs’, first-attempt ABS pass rate, comprehensive faculty evaluation	**Step 1**: Rotation evaluations and ‘drop outs’ were not associated with Step 1 scores. Residents with Step 1 score above mean had higher first-attempt ABS pass rate. Moderate correlation between Step 1 score and faculty evaluations (r = .28, p = 0.001).
					**Step 2**: N/A.
83	Predicting and enhancing American Board of Surgery In-Training Examination performance: does writing questions really help?	Willis et al	2016	ABSITE scores	**Step 1**: Step 1 score is significantly associated with ABSITE scores (p < 0.001).
					**Step 2**: Step 2 score is significantly associated with ABSITE scores (p < 0.001).
84	Predicting Performance on the American Board of Surgery Qualifying and Certifying Examinations	De Virgilio et al	2010	First-attempt pass rates on ABS qualifying and certifying examinations	**Step 1**: Scoring less than 200 on Step 1 significantly associated with failing both qualifying and certifying examinations (Odds Ratio of 0.36 for qualifying and 0.62 certifying).
					**Step 2**: N/A.
85	Predicting Success of Preliminary Surgical Residents: A Multi-Institutional Study	Al Fayyadh	2016	Resident obtaining first-choice categorical position after completing preliminary surgical training (primary success), resident obtaining any categorical position after completing preliminary surgical training (secondary success)	**Step 1**: No significant association.
					**Step 2**: Step 2 score significantly associated with primary success (p < 0.03) and secondary success (p < 0.02).
86	Relationships between study habits, burnout, and general surgery resident performance on the American Board of Surgery In-Training Examination	Smeds et al	2017	ABSITE Scores	**Step 1**: Residents scoring in the highest ABSITE quartile had significantly higher Step 1 scores (P < 0.001).
					**Step 2**: Residents scoring in the highest ABSITE quartile had significantly higher Step 2 scores (P < 0.001).
87	SCORE-Based Simulated ABSITE Exam Performance as a Predictor of Performance on the ABSITE	Shebrain et al	2021	ABSITE Score	**Step 1**: Small correlation between percent correct on ABSITE and Step 1 (r = 0.04, p = 0.653).
					**Step 2**: Small correlation between percent correct on ABSITE and Step 2 (r = 0.12, p = 0.16).
88	The Predictive Value of General Surgery Application Data for Future Resident Performance	Alterman et al	2011	ABSITE Scores and Faculty Evaluations	**Step 1**: USMLE Step 1 Score was significantly associated with ABSITE score only (p < 0.001).
					**Step 2**: N/A.
89	Using United States Medical Licensing Examination® (USMLE) Examination Results to Predict Later In-Training Examination	Spurlock Jr. et al	2010	ABSITE Scores	**Step 1 and Step 2**: Step 1 and Step 2 CK scores significantly correlated with ABSITE scores.
	Performance Among General Surgery Residents				**Step 2**: Step 2 CK scores are superior predictors of ABSITE PGY1-5 scores, specifically in PGY3 and PGY5.
90	USMLE Scores and Clinical Rotation Role in Predicting ABSITE Performance Among Surgery Interns	Elkbuli et al	2019	ABSITE Scores	**Step 1 and Step 2**: Step 1 and Step 2 CK both have significant correlation with ABSITE scores.
					**Step 2**: Step 2 has a stronger correlation with ABSITE scores (r = 0.44, P < 0.05).
91	What predicts surgical internship performance?	Andriole et al	2004	PGY-1 Program Director Performance Evaluations	**Step 1**: No significant association.
					**Step 2**: Step 2 has significant first-order association with PGY-1 PD performance evaluations (P < 0.01. Under multiple linear regression Step 2 score was the only predictor of PGY-1 PD performance evaluations.
**UROLOGY**					
92	Predictors of a Successful Urology Resident Using Medical Student Application Materials	Thompson et al	2017	Program Director Performance Evaluations	**Step 1**: No significant association.
					**Step 2**: Step 2 CK score was significantly associated with higher program director performance evaluation (P = 0.011).

*If Step 1 or Step 2 CK were not examined in a given study, the value ‘N/A.’ was assigned in the Data/Results column

**Table 2:** USMLE Step 1 and/or 2 CK Correlation with Residency Performance; Stratified by Performance Measure

Performance Measure	Article No.(s) demonstrating Step 1 was predictive	Article No.(s) demonstrating Step 2 CK was predictive	Article No.(s) demonstrating Step 1 was not predictive	Article No.(s) demonstrating Step 2 CK was not predictive
Board Certification	1, 3, 15, 20, 21, 27, 30, 31, 33, 40, 45, 46, 51, 60, 61, 64, 68, 69, 70, 71, 74, 76, 82, 84	3, 13, 15, 17, 21, 27, 30, 32, 35, 46, 51, 57, 60, 63, 69, 70, 71, 74, 75, 76	13, 32, 35, 58, 75	29, 34, 58, 64
In-Training Examination	3, 4, 7, 9, 18, 19, 24, 26, 32, 36, 38, 39, 42, 47, 52, 59, 65, 66, 72, 77, 79, 80, 83, 86, 87, 88, 89, 90	2, 3, 9, 18, 19, 24, 26, 29, 32, 41, 42, 43, 44, 52, 59, 65, 66, 80, 83, 86, 87, 89, 90	8, 37, 43, 44, 45	8
ACGME core competency/subcompetency milestone evaluations	12 (MK-01 only), 14, 22	12 (selected), 22	5, 10, 18, 23, 62	5, 14, 18, 23, 29
Faculty and Program Director Evaluations	49, 53, 55, 79, 82	48, 53, 55, 91, 92	48, 49, 88, 91, 92	49

*Only performance measures analyzed by≥ 3 studies are shown

## Results

A total of 1803 articles were screened from three separate databases. After excluding duplicates, irrelevant sources, and unoriginal research, 135 potential studies were identified from the titles and abstracts. A final screening, which included a full-text analysis, was conducted to determine eligibility. The 92 included studies were stratified by specialty, with Surgery (21.7% [20/92]), Emergency Medicine (13.0% [12/92]), Internal Medicine (10.9% [10/92]), and Orthopedic Surgery (8.7% [8/92]) being the most common. Results from each ACGME specialty were summarized in narrative format and specifics from every included study were listed (Tables 1 and 2).

### Anesthesiology

Study numbers (No.) 1–3, as referenced from Table 1, were specifically relevant to Anesthesiology resident performance. Performance measures assessed across the three studies included Anesthesiology board certification, ITE scores, and Anesthesiology Knowledge Test (AKT) ranking. For board certification, No. 1 and 3 showed that Step 1 was predictive, and No. 3 showed that Step 2 CK was predictive. For ITE scores, No. 3 showed that Step 1 was predictive, and No. 2 and 3 showed that Step 2 CK was predictive. Additionally, No. 3 demonstrated that both Step 1 and Step 2 CK were correlated with AKT ranking. In summary, both Step 1 and Step 2 CK were predictive indicators of resident performance.

### Dermatology

Study No. 4 was specifically relevant to Dermatology resident performance. In this study, Dermatology ITE-1, ITE-2, and ITE-3 scores were assessed as the outcome measure. After analyzing their results, the authors suggested that Step 1 scores correlated with ITE scores for each year of residency. After the screening, no data was found regarding Step 2 CK and Dermatology-specific resident performance measures.

### Emergency medicine

Studies No. 5–16 were specifically relevant to Emergency Medicine resident performance. Performance measures assessed across these studies included Emergency Medicine board certification, ITE scores, ACGME core competency milestone evaluations, Script Concordance Test (EM-SCT) scores, measured negative resident outcomes, and resident graduation rank order list (ROL). For board certification, No. 15 showed that Step 1 was predictive, while No. 13 declared that Step 1 was not predictive. Both No. 13 and 15 showed that Step 2 CK was predictive. For ITE scores, No. 7 and 9 claimed that Step 1 was predictive, while No. 8 suggested Step 1 was not predictive in a multivariable linear regression model. Study No. 9 showed that Step 2 CK was predictive, while No. 8 claimed that Step 2 CK was not correlated with the ITE score in a multivariate linear regression model. Regarding ACGME milestone evaluations, results were generally mixed concerning Step 1 and Step 2 CK predictive value as demonstrated in No. 5, 10, 12, and 14. Study No. 6 showed that Step 2 CK correlated with the EM-SCT score. Study No. 11 demonstrated that Step 1 was predictive of measured negative resident outcomes. the resident ROL was not correlated with the Step 1 score according to No. 16. In summary, the predictive value of Step 1 and Step 2 CK was mixed and was largely dependent on the specific outcome measure being assessed.

### Family medicine

Studies No. 17 and 18 were specifically relevant to Family Medicine resident performance. Performance measures assessed across the two studies included Family Medicine board certification, ITE scores, and ACGME core competency milestone evaluations. For board certification, No. 17 showed that Step 2 CK was predictive. For ITE scores, No. 18 showed that both Step 1 and Step 2 CK were predictive. However, No. 18 demonstrated no correlation between either Step score and ACGME milestone evaluations. In summary, Step 1 and Step 2 CK was associated with examination scores while no association was found with ACGME milestone ratings.

### Fellowship - hematology and medical oncology

Studies No. 19 and 20 were specifically relevant to Hematology and Medical Oncology fellow performance. Performance measures assessed across the two studies included board certification, ITE scores, and the number of awards, abstracts, and publications during the fellowship. For board certification, No. 20 showed that Step 1 was predictive. For ITE scores, No. 19 demonstrated that both Step 1 and Step 2 CK were predictive. However, No. 19 indicated that neither Step 1 nor Step 2 CK was associated with the award, abstract, or publication number during the fellowship. In summary, Step 1 and Step 2 CK was associated with examination scores while no association was found with the number of awards, abstracts, and publications.

### Fellowship - infectious disease

Study No. 21 was specifically relevant to Infectious Disease fellow performance. In this study by Grabovsky et al., the outcome measures assessed were ABIM-ID certification examination score and ABIM-ID certification examination passing status. The authors found that the Step 1 score correlated with the ABIM-ID certification examination score, while Step 2 CK was associated with ABIM-ID certification examination passing status.

### Fellowship - neuroradiology

Study No. 22 was specifically relevant to Neuroradiology fellow performance. In this study by Yousem et al., the outcome measures assessed were Fellow E*Value scores (determined by faculty assessment of ACGME core competencies) and best-to-worst ranking within the fellowship cohort. After analyzing their results, the authors claimed that both Step 1 and Step 2 CK showed predictive value concerning the identified performance measures.

### Internal medicine

Studies No. 23–32 were specifically relevant to Internal Medicine resident performance. Performance measures assessed across these studies included Internal Medicine board certification, ITE scores, ACGME core competency milestone evaluations, structured clinical exam performance, long block 360-degree ratings, direct patient assessment of physician attributes, and performance ratings. For board certification, No. 27, 30, and 31 showed that Step 1 was predictive, while No. 32 showed that it was not predictive. Studies No. 27, 30, and 32 claimed that Step 2 CK was predictive, while No. 29 demonstrated no predictive value. For ITE scores, No. 24, 26, and 32 showed that Step 1 was predictive, and No. 24, 26, 29, and 32 showed that Step 2 CK was predictive. For ACGME competency milestone evaluations, Step 1 was not a predictor according to No. 23, and Step 2 CK showed no correlation in No. 23 and 29. Study No. 25 showed that neither Step 1 nor Step 2 CK was associated with structured clinical exam performance. For long block 360-degree ratings, Step 2 CK was predictive while Step 1 was not according to No. 32. That same study showed that Step 2 CK, but not Step 1, was associated with direct patient assessment of physician attributes. Lastly, No. 28 demonstrated that Step 1 had predictive value for performance ratings. In summary, the predictive value of Step 1 and Step 2 CK was mixed and was largely dependent on the specific outcome measure being assessed.

### Neurological surgery

Out of all screened articles across three databases, studies No. 33 and 34 were found to be pertinent to Neurological Surgery resident performance. In Neurological Surgery, the main performance measure being assessed by the studies was the American Board of Neurological Surgery (ABNS) scores. The studies were not in conflict with their conclusions, with No. 33 stating that Step 1 was correlated with ABNS scores and No. 34 stating that Step 2 CK was not correlated with ABNS scores. In summary, Step 1 correlated with resident performance while Step 2 CK did not.

### Obstetrics and gynecology

In Obstetrics and Gynecology studies No. 35 and 36 were found with relevant results. Step 2 CK scores were found in No. 36 to be correlated with board certification and separately Step 1 scores were found by No. 35 to be significantly correlated with Council on Resident Education in Obstetrics and Gynecology in-training examination scores. Together, Step 1 and Step 2 CK were found to be correlated with at least one measure of resident performance in the field of Obstetrics and Gynecology. It should be noted that Step 1 had no association found with board certification, indicating a more limited application.

### Ophthalmology

Studies No. 37–39 were found with relevant information in the field of Ophthalmology. The main performance indicators looked at for Ophthalmology were the Ophthalmic Knowledge Assessment Program (OKAP) exam and the American Board of Ophthalmology written qualifying examination (ABO-WQE). The studies were all concerned with how Step 1 impacted these performance measures and they were decidedly disparate in their conclusions thereby precluding any clear-cut larger takeaways. In summary, the findings indicate mixed results and no conclusion can be found concerning a correlation of Step 1 scores to resident performance.

### Orthopedic surgery

Studies No. 40–47 were found with relevant information. Performance measures assessed included the American Board of Orthopedic Surgery (ABOS) Certifying Examination and Orthopedics In-Training Exam (OITE) scores. The ABOS scores reported that both Step 1 and Step 2 CK had a positive correlation. For OITE scores it was agreed upon in the publications that Step 2 CK had a positive correlation, but there was disagreement over whether the same could be said of Step 1. In summary, Step 2 CK positively correlated with both OITE and ABOS as indicators of resident performance, and Step 1 only correlated with ABOS.

### Other - multispecialty residency publications

Studies No. 48–58 looked at the correlation of Step 1 and/or Step 2 CK scores to resident performance across multiple specialties. These studies generally fell into two types: those that used surveys of residency directors on resident performance as a measure, or those that used broad board exam certification rates as a measure. There is a significant amount of perceived conflict in the findings of these broader studies. The finding suggests that Step 1 and Step 2 CK had some correlation with board certification rates. It also seems that Step 1 was not correlated with professionalism as a resident. In general, these multispecialty residency publications ultimately lack cohesive conclusions about Step 1 or Step 2 CK as indicators of resident performance.

### Otolaryngology - head and neck surgery

The main performance metrics found in the literature were OTE scores and WQE passage. Only studies No. 59 and 60 were found concerning Otolaryngology, and they did not conflict in their viewpoint that Step 1 and 2 CK both correlated with OTE and WQE performance. In conclusion, both Step 1 and Step 2 CK had solid positive correlations with resident performance.

### Pathology

Pathology only had study No. 61 identified; a piece that found Step 1 to be correlated with ABP passage/failure rate. Therefore, the only conclusion that can be made in this specialty is that Step 1 was positively correlated with resident performance.

### Pediatrics

Studies No. 62–64 identified those that could provide findings on Step 1 and Step 2 CK correlation with resident performance. Board passage rate and residency milestone scores were used as indicators of success. Step 1 was not found to be correlated with milestone scores, while it did correlate with board passage rates. For Step 2 CK, the findings conflicted as related to board passage rates. In conclusion, Step 1 and Step 2 CK predictive values were mixed for pediatric resident performance measures.

### Psychiatry

Studies No. 65–66 were identified for psychiatry utilizing Psychiatry Resident-In-Training Examinations (PRITE) and psychotherapy treatment session evaluations as performance measures. Both studies noted significant correlations between Step 1 and Step 2 CK scores and resident performance based on these measures.

### Radiology

Studies No. 67–71 were identified for Radiology utilizing American Board of Radiology (ABR) core examination scores, rotation evaluations, retrospective faculty recall scores, and cumulative major discordance rate for on-call cases as performance measures. Step 1 showed mixed correlation with resident success, with higher scores predicting lower major discordance rates in No. 67 and better ABR core examination performance in No. 68–71, but not predicting rotation performance. Step 2 CK consistently showed a positive correlation, with higher Step 2 CK scores indicating better ABR core examination performance in No. 69–71. Overall, Step 1 showed mixed success as an indicator of radiology resident performance while Step 2 CK was a significant indicator of resident performance.

### Surgery

Studies No. 72–91 were identified in the Surgery specialty. Out of the 20 articles identified, American Board of Surgery In-Training Examination (ABSITE) scores, American Board of Surgery Qualifying and Certifying Examinations performance, faculty evaluations, Emotional Intelligence (based on Trait EI Questionnaire), resident remediation and attrition rates, categorical position placement, resident awards, case logs, scholarly activity, and professionalism were all utilized as performance measures. Step 1 showed a mixed correlation with surgical resident success. With few exceptions, many studies on objective performance measurements (e.g., In-Training Examinations, Qualifying and Certifying Examinations, remediation, and attrition), such as No. 72, 74, 76, 77, 79–84, and 86–90, showed a significant positive correlation with Step 1 scores. However, No. 73 and 91, which analyzed more subjective measures of resident performance (e.g., faculty evaluations), showed no correlation or even negative correlation in the case of resident awards. Step 2 CK also showed a mixed correlation with surgical resident success but showed a more positive correlation with overall resident performance than Step 1 demonstrated in No. 89 and 90. Step 2 CK scores showed a positive correlation with some of the same objective measures Step 1 did (e.g. In-Training Examinations, Qualifying, and Certifying Examinations), but went further to show a positive correlation with numerous other measures including Emotional Intelligence in No. 78, resident obtaining a first-choice categorical position in No. 85, and program director evaluations in No. 91. In summary, Step 1 and Step 2 CK both showed mixed correlation with surgical resident performance, with Step 2 CK showing a slightly more comprehensive correlation.

### Urology

One relevant study, No. 92, was identified for Urology, utilizing Program Director Performance Evaluations as the performance measure. This study showed that Step 2 CK was significantly associated with higher Program Director Performance Evaluations, while Step 1 was not.

## Discussion

### Scope of literature

There have been various studies on how Step 1 and Step 2 CK scores correlate with medical resident performance but these studies have tended to be specialty-specific and varied greatly by performance measurement. This systematic and comprehensive review is the first to our knowledge considering the use of Step 1 and Step 2 CK as predictors of comprehensive resident performance across all ACGME-accredited specialties. Considering the immense weight placed on USMLE Step 1 and Step 2 CK success, only 92 studies relevant to this systematic review have been performed since the birth of these exams in the early 1990s. Taken together, this identifies an insufficient investigation by the medical community into their true predictive validity.

The scope of literature currently available regarding the utility of Step 1 and Step 2 CK as comprehensive indicators of resident performance can be appreciated in specific detail in both Tables 1 and 2. Areas, where either or both of the Step exams have not had any findings for a major performance measure in a given specialty, should be considered prime targets for further investigation. Results from subsequent investigations based on the gaps indicated by this systematic review will do a service to the program directors and other leaders in those specialties that finally receive overdue attention from the literature on residency performance prediction.

### Step 1 and step 2 CK as performance predictors

The discussion regarding Step 1 and Step 2 CK as indicators of resident performance is a complicated one for two major reasons. First, it is important to recognize the potential possibility that not all medical residencies are equivalent. Therefore, to control for any potential differences concerning the field of study, the results of the systematic review were interpreted by initially stratifying the performance data by resident specialty in Table 1. Second, a case can be made that “resident performance” is an umbrella term that encompasses several different sub-categories including Board Certification, In-Training Examinations, ACGME core competency evaluations, Faculty and Program Director evaluations, and many others. Table 2 was constructed to appreciate the possibility that the USMLE Step exams may correlate to certain outcome measures and not others.

To begin the analysis, Steps 1 and 2 CK as performance indicators will be discussed for the first organizational strategy: *Specialty*. Steps 1 and 2 CK both demonstrated predictive value for resident performance outcomes in Anesthesiology, Infectious Disease and Neuroradiology fellowships, Otolaryngology-Head and Neck Surgery, and Psychiatry. Only Step 1 showed predictive value for resident performance outcomes in Dermatology, Neurological Surgery, and Pathology, while only Step 2 CK showed predictive value for resident performance outcomes in Obstetrics and Gynecology, Orthopedic Surgery, Radiology, and Urology. Both Step 1 and Step 2 CK showed mixed results for several specialties including Emergency Medicine, Family Medicine, Hematology and Medical Oncology Fellowship, Internal Medicine, Ophthalmology, Pediatrics, and Surgery. It is imperative to keep in perspective that these results are limited by the fact that relevant literature was lacking for many specialties. Some specialties had zero relevant articles, while certain others had one or two. More research should be conducted to assess the reproducibility of the results and strengthen the confidence in conclusions made for specialties lacking robust data.

To continue the analysis, Steps 1 and 2 CK as performance indicators will be discussed concerning the second organizational strategy: *Outcome measure*. An interpretation of Table 2 allows us to make generalized statements for both USMLEs. Importantly, an outcome measure was included in Table 2 only if three or more studies assessed it to increase confidence in the general conclusions that were made. Both Step 1 and Step 2 CK showed predictive value for Board Certification during residency across several different studies, with a few exceptions noted. Concerning In-Training Examinations, many articles demonstrated that Step 1 had predictive value, with a few exceptions noted. Step 2 CK also demonstrated strong predictive value, with only one exception noted. Using ACGME core competency milestone evaluations as the performance measure, Step 1 and Step 2 CK showed no predictive value, however notable exceptions to this generalization are shown in Table 2. Regarding Faculty and Program Director evaluations, Step 1 predictive value was mixed, while Step 2 CK showed predictive value in all relevant publications except one. It is important to consider the limitation that these conclusions from Table 2 are drawn across multiple studies assessing several diverse specialties and therefore may not account for any potential confounding variables associated with inherent differences between medical residencies.

The results of this study have merit and should be contemplated in the context of the decision to make Step 1 a pass/fail examination. On the one hand, Step 1 showed positive correlative value for certain outcome measures, particularly those related to standardized test-taking ability. On the other hand, Step 2 CK can largely provide much of the same information about resident performance, and perhaps even more so (i.e., predictive for Faculty/Program Director Evaluations). One downside of Step 2 CK remaining as a numeric score will be the inevitably increased pressure on medical students to perform well on this single examination.

The results of this systematic review demonstrate that both Step 1 and Step 2 CK can be useful, at least in some respects, across a variety of resident specialties and performance measures. In other respects, their value is either not researched enough or not replicable across the publications analyzed here. It is the opinion of the authors that these examinations, while helpful in some cases, should only be used as tools in the holistic assessment of future performance in residency. “Performance” is quite complex in its definition, particularly concerning a medical resident, and therefore a wide variety of assessment methods should be considered.

## Limitations

It is important to interpret these findings in the context of limitations. Importantly, some of the resident indicators analyzed may be inherently unreliable as measures of overall resident success. Low correlations and negative findings may be due to characteristics of the resident performance measures and not due to the predictive power of USMLE scores. Additionally, many of the studies cited used terminology such as “predictor”, “association”, or “indicator” to describe the relationship between USMLE scores and resident performance metrics. It is important to note that this use of ‘predictor’ or ‘indicator’ does not imply causation and is only a description of positive association.

The findings from this study also should be understood to be of limited value in comprehending the true clinical proficiency of a given physician outside of the context of resident evaluation metrics. The term “performance” should not be misconstrued as referring to this wider authentic clinical ability which our study has not established as being linked to step 1 and 2 scores. Our findings should be most applicable to residency program directors and other organizers who would like a better understanding of the true association between step scores and resident performance metrics. That being said, there is a heterogeneity that must be acknowledged in residency program structure, quality, and educational emphasis that is reflected in the wide range of metrics that were relied upon in studies collected within this review.

Furthermore, it is critical to acknowledge that “objective measures” and “standardization” do not guarantee a lack of bias. Some standardization procedures may be inadvertently influenced by structural determinants that may disadvantage certain groups. Given this, it is essential for future research to explore these potential biases to ensure fairness and equitable evaluation.

Additonailly it is important to note that we have chosen not to utilize tools such as the Medical Education Research Study Quality Instrument (MERSQI) and the Newcastle-Ottawa Scale for Education (NOS-E) in the analysis of our findings for two main reason. First, the primary objective of our study was to conduct a systematic review and synthesis of the existing literature in order to investigate any possible relationship(s) between Step 1/2 performance and resident outcomes across different medical specialties. While the inclusion of quality assessment tools can be valuable, our intention was to include all available data that passed our screening process. We did not intend to rank or weigh each study based on its methodology. Second, assessing the quality of studies in the field of medical education and residency performance prediction may be possible, but is it is a complex task. The use of tools like MERSQI and NOS-E requires a detailed evaluation of various aspects of study design, methodology, and reporting. Applying these tools retrospectively to a wide range of studies with diverse research designs, settings, and outcome measures was outside the scope of our core objective.

## Conclusions

These studies have reported value and are imperative to discern the utility of Step 1 and Step 2 CK as predictors of resident performance and as tools for resident recruitment and selection. The results of this systematic review suggest that both a scored Step 1 dated before January 2022 and Step 2 CK can be useful as tools in a holistic review of an applicant to residency programs. Given its inherent complexity, multiple tools across many assessment modalities are necessary to assess resident performance. Future research should explore the combined predictive value of standardized USMLE examinations, clinical evaluations, holistic review practices, and other critical skills to evaluate a more comprehensive approach to evaluating residency candidates.

## Electronic supplementary material

Below is the link to the electronic supplementary material.

Supplementary Material 1

## Data Availability

All data generated or analyzed during this study are included in this published article and its supplementary information files.

## Abbreviations

USMLE: United States Medical Licensing Examination
CK: Clinical Knowledge
CS: Clinical Skills
NBME: National Board of Medical Examiners
FSMB: Federation of State Medical Boards
PRISMA: Preferred Reporting Items for Systematic Reviews and Meta-Analyses
