# Supplementary material for: USMLE step 1 and step 2 CK as indicators of resident performance
Source: BMC Med Educ. 2023 Jul 31;23:543. doi: 10.1186/s12909-023-04530-8 (PMC10391769; doi:10.1186/s12909-023-04530-8)
Supplement: Supplementary file 1 — Supplementary Material 1 [file 12909_2023_4530_MOESM1_ESM.docx]

**Appendix E1.** Complete search strategy utilized to survey MEDLINE and adapted for Cochrane Library and Embase**.**

**
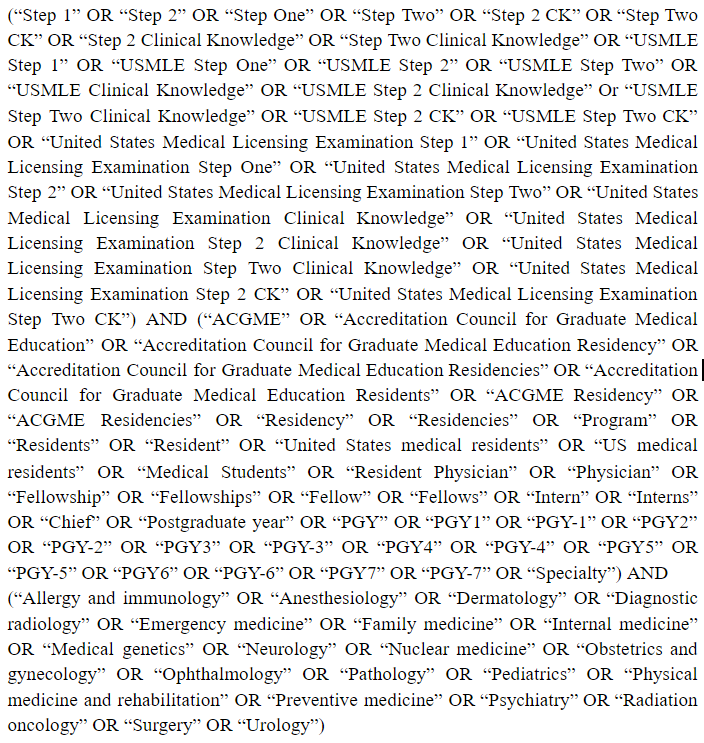
**
